# GHRH-GH-IGF1 axis in pediatric Down syndrome: A systematic review and mini meta-analysis

**DOI:** 10.3389/fped.2023.1132296

**Published:** 2023-02-22

**Authors:** David Shaki, Eli Hershkovitz, Shai Tamam, Arkadi Bollotin, Odeya David, Guy Yalovitsky, Neta Loewenthal, Lior Carmon, Dganit Walker, Alon Haim

**Affiliations:** ^1^ Pediatric Endocrinology Unit, Soroka University Medical Center, Beer Sheva, Israel; ^2^ Faculty of Health Sciences, Ben-Gurion University, Beer Sheva, Israel; ^3^ Library of Life Sciences and Medicine, Tel Aviv University, Tel Aviv, Israel

**Keywords:** growth hormone, Down syndrome, IGF1, neurosecretory dysfunction, bio-inactive GH, hypothalamus

## Abstract

**Objective:**

To analyze and determine the quality of functioning in different components of GHRH-GH-IGF1 axis in children with Down syndrome (DS).

**Design:**

Systematic review and mini meta-analysis of the literature.

**Methods:**

A search was performed in PubMed, Embase, Scopus, and PsycINFO through August 2022. Eligible studies included pediatric patients with DS who had undergone any laboratory evaluation of the GHRH-GH-IGF1 axis. Two reviewers independently screened articles for eligibility. Results of each type of test were weighed together in patients both with and without DS and were pooled using a random effects meta-analysis.

**Results:**

In total, 20 studies assessed the GHRH-GH-IGF1 axis function. A defect in three major components of GHRH-GH-IGF1 axis was found in a significant proportion of pediatric DS patients.

**Conclusions:**

A significant portion of short-stature pathogenesis in children with DS is associated with impaired GHRH-GH-IGF1 axis function.

## 1. Introduction

Down syndrome (DS) is the most common chromosomal disorder with an incidence of one in 700 live births in the United States (1), and 1–10 in 1,000 live births worldwide, according to the WHO (2). Linear growth retardation is a cardinal characteristic of DS. Pathologic low height velocity is most marked in infancy and adolescence (3). The mechanisms responsible for short stature in DS are not yet completely clear. Although congenital heart defects may contribute to growth retardation, short stature characterizes children with DS even in the absence of such defects.

Various hypotheses about its cause have been raised over the years. The main pathogenic route examined is the existence of interference in one of the “stations” along the GHRH-GH-IGF1 axis. The GHRH-GH-IGF1 axis consists of a chain of events that start with the release of growth hormone-releasing hormone (GHRH) from the hypothalamus. GHRH then travels to the pituitary gland, where it stimulates the release of growth hormone (GH). GH then travels to the liver, where it stimulates the production of insulin-like growth factor 1 (IGF-1). IGF-1 is a hormone that plays a critical role in growth and development.

The involvement of GHRH-GH-IGF1 axis in the pathogenesis of short stature in DS children is strongly suggested by growth retardation having been most marked between the ages of 6 and 24 months, the same period that growth hormone normally becomes the main regulator of growth (4).

Various studies have reported different results of tests that assessed the axis function, usually on a small number of patients. Therefore, it has been difficult to reach a valid conclusion on this matter.

In this study, for the first time, we reviewed all the reported data that examine the possible disruption in the GHRH-GH-IGF1 axis in DS pediatric patients.

## 2. Methods

### 2.1. Search strategy and study selection

The present systematic review was performed in accordance with the Preferred Reporting Items for Systematic Reviews and Meta-Analyses (PRISMA) guidelines (5). No formal ethical approval was required. An extensive literature search of four electronic databases: PubMed, Cochrane Library, Scopus, and PsycINFO *via* EBSCO was undertaken for studies about DS and growth hormone, published until January 2021. The general keywords were “Down syndrome” and “growth hormone”, while the search strategy was updated and adapted for each database. The search was restricted to humans, and no other restriction was made. Studies in all languages were included. Full-text articles of potentially relevant studies not available through the university library were requested from the authors. We ran a repeat search on 01.09.2022 and received 27 additional records. A review of the title or abstract was enough to determine that they are not suitable for inclusion in this review.

### 2.2. Quality assessment and risk of bias

The scope of data reporting in a large part of the original works did not allow for a full quality and risk of bias assessment to be carried out on the individual original studies, therefore no individual quality assessment was carried out.

We used ROBIS, a tool for assessing quality and risk of bias in systematic reviews. The tool is completed in three phases: assess relevance (optional), identify concerns with the review process by 21 questions divided to 4 domains, and judge risk of bias in the review. It is the first rigorously developed tool designed specifically to assess the risk of bias in systematic reviews (6).

### 2.3. Eligibility criteria

**Figure 1:**
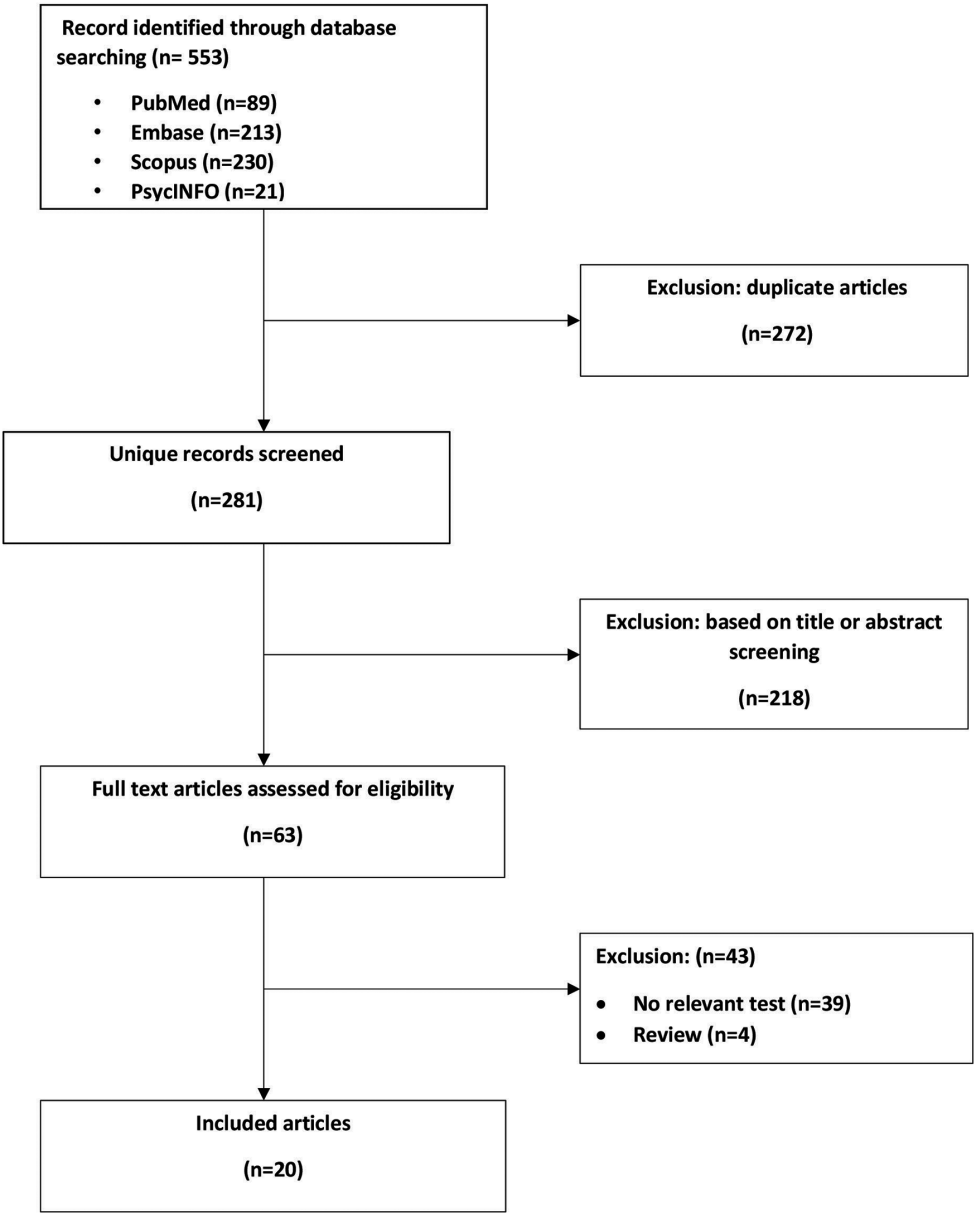
Flowchart of article screening and inclusion.

Eligible studies included focused on the assessment of the GHRH-GH-IGF1 axis function in pediatric DS patients and reported results of at least one of the following tests: growth hormone stimulation test, 12- or 24-hour integrated GH concentration test, IGF-1 level assay, IGF-1 generation test, calculated bind GHBP (growth hormone binding protein)/ total GHBP ratio, or calculated GH Radioreceptor assay (RRA) / immunoradiometric assay (IRMA) ratio. Both comparative as well as single-arm studies were considered, as were single case reports. Figure 1 describes the flowchart of article screening and inclusion.

### 2.4. Data extraction

Data extraction included a full description of participants enrolled including their gender and age. Foreign language articles were translated by multilingual reviewers. The main outcome extracted from studies for mini meta-analysis was the percentage of patients with pathologic test results.

### 2.5. Statistical analysis

In order to assess heterogenicity, we used a random effects meta-analysis. We assessed the degree of inconsistency in the results between studies using the I² statistic. This statistic explains the proportion of inconsistency between studies that cannot be explained by chance alone and is likely due to real differences in the population or the conduct of the studies (7).

Results of each type of test were weighed together in patients both with and without DS and were pooled using a random effects meta-analysis. We used a Chi-square test to evaluate the differences between test results of DS patients with those of control groups. Publication selection bias could not be calculated due to missed relevant data.

## 3. Results

### 3.1. Search results

An initial search of the literature yielded 281 publications and 20 eligible studies (Figure 1). Twenty studies examined one or more aspects of the GHRH-GH-IGF1 axis function in children with DS. Eight of the studies also included a control group of children without DS. The main findings are presented in Table 1.

**Table 1:** GHRH-GH-IGF1 axis mini meta-analysis—main findings of included studies.

Lead Author (Year)(Ref)	Participants with DS	Normal Controls	Age	Tests	Main findings
Milunsky A (1968) (8)	7	7	2–6 (y)	Insulin test	Pathological 0%
Pozsonyi J (1971) (9)	12	–	0.6–20 (m)	A–Fasting GH level B–Insulin test-(12) C–Arginine test-(4)	A–Noticeably low levels (before the arginine test—0.1 compared to the other participants—developmental delay for another reason) B–Peak average 12.78 Ng/ml, one pathological C–Peak average 7.88 Ng/ml, one pathological
Ruvalcaba RH (1972) (10)	7		8–11 (y)	Arginine test	14% pathological
Visci R Sara (1983) (11)	21		0–11 (y)	IGF1 and IGF2 levels	IGF1 level is above normal in the first 2 years of life but fail to increase with advancing age, therefore over the years becomes pathological low. IGF2 level is elevated throughout life.
Annerén G (1986) (12)	5		3–6(y)	A–Insulin-arginine test B–2 h Sleep test C– IGF1 generation test	A–20% pathological B–60% pathological C–All were below the norm at baseline, and everyone entered the norm after 10 days
Annerén G (1990) (13)	5	-	3.6–6 (y)	A–RIA IGF1 levels B–RIA IGF2 levels C–RRA IGF total levels	A–2 years old—lower than normal and no later recovery B–Normal throughout life C–identifies both IGF1 and IGF2 and fetal form, beginning with post-natal period—high throughout life
Torrado C (1991) (14)	13		1–5(y)	A–Clonidine test B–L Dopa test	A–61.5% pathological B–61.5% pathological
Castells S (1992) (15)	20	7	1–13(y)	A–Clonidine test B–L Dopa test C–Targeted concentration 12/24 h.	A–65% pathological B–60% pathological C–100% pathological
Pueschel SM (1993) (16)	8		1–6 (y)	A–Clonidine test B–L Dopa test C–GHRH test	A–50% pathological B–37.5% pathological C–12.5% pathological
Barreca A (1994) (17)	18	8	1–11 (y)	A–IGF1, IGF2 level-basal (39 patients) B–Arginine test C1–IGF1 levels 12,24 and 48 h. after arginine injection C2–IGF1 levels 12,24 and 48 h. after GH injection. D–GH RRA/ IRMA ratio E–Bind GHBP/total GHBP ratio (DS patients vs. non-DS control)	A–Normal IGF2 level. Pathological low IGF1 level at 36% (GR1-low basal IGF1 concentration). In remaining patients (GR2-IGF1 in the low-normal range) B–GR1-peak average 29.6 Ng/ml. GR2-peak average 15.1 Ng/ml C1–GR1-no significant increase GR2-significant increase in IgF1 level C2–GR1—a lower peak compared to GR2, but a larger delta D–Ratio is lower in GR1—an expression of GH bioactivity. The difference in relation was on the signiﬁcance boundary with *p* = 0.057. Especially in 2 patients, a very low level. Statistically significant correlation between the ratio and IGF1 level, and IGF1 peak after the arginine test. E–Similar ratio between groups
Castells S (1996) (18)	14	7	1–5 (y)	A–Clonidine test B–L Dopa test C–GHRH test	A–Pathological 50% B–Pathological 43% C–Pathological 0%
Castells S (1996) (19)	40		prepubertal	A-IGF1 B-L-DOPA test C- CLONIDINE test D-GH overnight test (2 children only)	A- an average of 65 ng/ml per square, which is slightly above the lower border of the norm. B-at least 35% pathological, maximum 95% vs. 0% pathological in the control group C-at least 38% pathological to 100% maximum vs. 0% pathological in the control group D-one patient was tested after 2 normal tests, a pathological result.
Ragusa L (1996) (20)	40		1–19 (y)	A-clonidine test B-Insulin test C -if discordant results—24-hr integrated concentration test) D- GHRH plus pyridostigmin test E-IgF1 levels in those with at least 1 pathological test (clonidine or insulin)	A-pathological 62.5% B-pathological 40% C-integrative concentration test 24 h – 83.3% Pathological D-GHRH plus pyridostigmin 30% received pathological E- 43% pathological, And another 38% below average (25% below quarter, 15% below quarter, 10% below decile)
Proto C (1997) (21)	31		1–20 (y)	A-IgF1 LEVEL B- IGFBP3 level	A-out of 18 DS children without growth hormone deficiency, in 5 IGF1 level was below the norm. Out of 13 with growth hormone deficiency, 6 had a low IGF1. B-only 3 had low IGFBP3 levels.
Ragusa L(1996) (22)	16		1.5–16.2 (y)	A-clonidine test B-insulin test C-GHRH test D-GHRH plus PD E-hexarelin	A-average 13.3 Ng/ml B-average 23.1 Ng/ml C-average 27.2 Ng/ml D-average 47.8 Ng/ml E- average 58.4 Ng/ml
Ferri R (1996) (23)	9	1	12.8 (y avg)	A–Compare average GH level between the various sleep stages B–Percentage of time spent in each sleep phase C–Number of GH secretion episode peaks D–Average peak amplitude E–Average peak duration F–Area under the curve of peaks alone G–Integrated concentration	A–A significant low level of average GH level on S3 + 4 sleep stages compared to control. B–A significant low percentage on S3 + 4 sleep stages compared to control. In A + B No gross difference during wakefulness or the different sleep stages in DS patients C–The average number of peaks is 2.6 D–Significantly low in DS compared to control E–Significantly low in DS compared to control F–Significantly low in DS compared to control G–Significantly low in DS compared to control
Arvat E (1996) (24)	15	15	13.5 &11.9 (Y, mean)	A–IGF1 level B–GHRH test C–GHRH plus PD test	A–Similar IGF1 level between DS children compared to normal child control B–Similar GH results between DS children compared to normal children- controls C–Similar GH results between DS children compared to normal children- controls
Ragusa L (1998) (25)	113	162	12–13 (y)	A– Average IGF1 Level in DS compared to control. B–percentage of children with IGF1 level below the norm for age and maturity level	A–Significant slightly lower (258 mcg/L vs. 311 mcg/L) B–15% below the norm.
Yasuhara A (2001) (26)	1		5(y)	A–IGF1 level B–Arginine test C–Glucagon test D–GHRH test E-2.5 h’ Sleep test	A–Very low B- Pick 22.8 Ng/ml C-pick 23 Ng/ml D- Pick 17 Ng/ml E- Pick 5 Ng/ml, average 3.3 Ng/ml
El Gebali H (2014) (27)	40	40	3–11 (y)	IgF1 level	A–No difference found between children with DS and healthy control

The percentage of pathological results among DS patients ranged between 0% and 25% in studies that examined the arginine stimulation test (9, 10, 17), 0% and 40% for insulin stimulation tests (8, 9, 20), 37.5% and 61.5% for L-DOPA stimulation tests (14–16, 18), and 50% to 65% for clonidine stimulation tests (14–16, 18, 20). Studies that examined the GHRH stimulation test with and without pyridostigmine found between 0% and 30% of DS patients with a pathological result (16, 18, 20) and 0% in the hexarelin stimulation test (22). Studies that examined the 12- or 24-hour integrated GH concentration test found between 83.3% and 100% of DS patients with a pathological result (15, 19, 20). Studies examining the level of IGF-1 in DS patients found it to be below the normal range for age and gender in 15% to 43% of the cases and below the 25th percentile for between 64% and 100% (17, 20, 21, 25). In the IGF-1 generation test among DS patients, while no pathologically low IGF-1 level was recorded for those given recombinant growth hormone (12, 17), 100% of a pathologically low IGF-1 level was recorded for those given arginine (17). In the bind GHBP/total GHBP ratio test among DS patients, 0% of a pathological result was found (17). However, in the RRA/IRMA ratio test the ratio was significantly lower in those with an IGF-1 level below the norm (17). The nocturnal GH peak characteristics, which include amplitude, duration, and area under the curve, was found to be significantly low for DS patients compared to the control group (23).

### 3.2. Mini meta-analysis GHRH-GH-IGF1 axis function

Risk of Bias of systematic review was evaluated according to ROBIS. Phase 2 include 3 essential domains: identification and selection of studies, data collection and study appraisal, synthesis, and findings. Thirteen signaling questions in three domains were corresponded to “low risk of bias” while 3 signaling questions, 2 in domain 3 and one in domain 4, were classified as “no information” and hence corresponded to “unclear risk of bias”.

**Figure 2:**
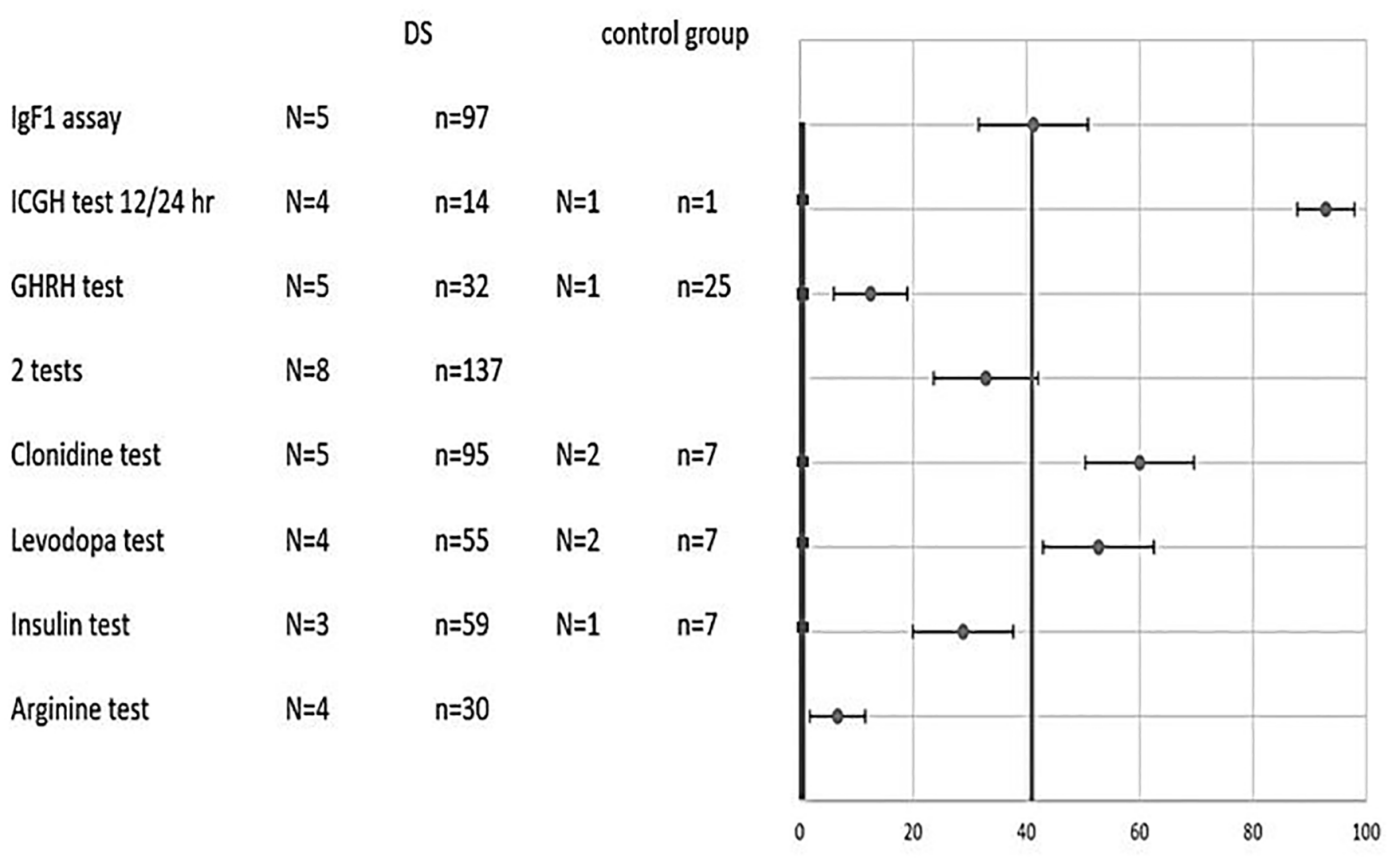
Combined results of various tests in DS and control groups. Percentage of pathological results (x-axis) for each test (y-axis) of DS patients (circle) vs. control (square). Two average lines that weigh all tests’ results (control-thicker line, DS- thinner line) are shown as well. N- number of studies. n-total number of subjects. 2 tests- subjects who undergo 2 different growth hormone stimulation tests.

Comparisons of pathological results in percentage terms between DS and non-DS children for each test as presented in Figure 2 were based on all studies from which the relevant data could be extracted. For the arginine test the overall percentage of pathological results among DS children was 7%, for the GHRH test - 12%, for the insulin test - 29%, for the L-DOPA test - 53%, for the clonidine test - 63%, and for the 12- or 24-hour integrated GH concentration test - 93%. Among DS patients undergoing the growth hormone stimulation testing protocol, 32% registered pathologically low results in two tests. IGF-1 levels were found to be pathologically low according to age and gender in 40% of DS patients. It was not possible to extract the percentage of pathological results in a small number of studies (11, 13, 22). The overall percentage of pathological result was 0% for all tests among the control groups.

Weighing the total results for all tests on DS children cumulatively yielded 40% pathological results compared to 0% in the non-DS control groups (Figure 2). The statistical difference is significant with *p* value <0.001.

**Figure 3:**
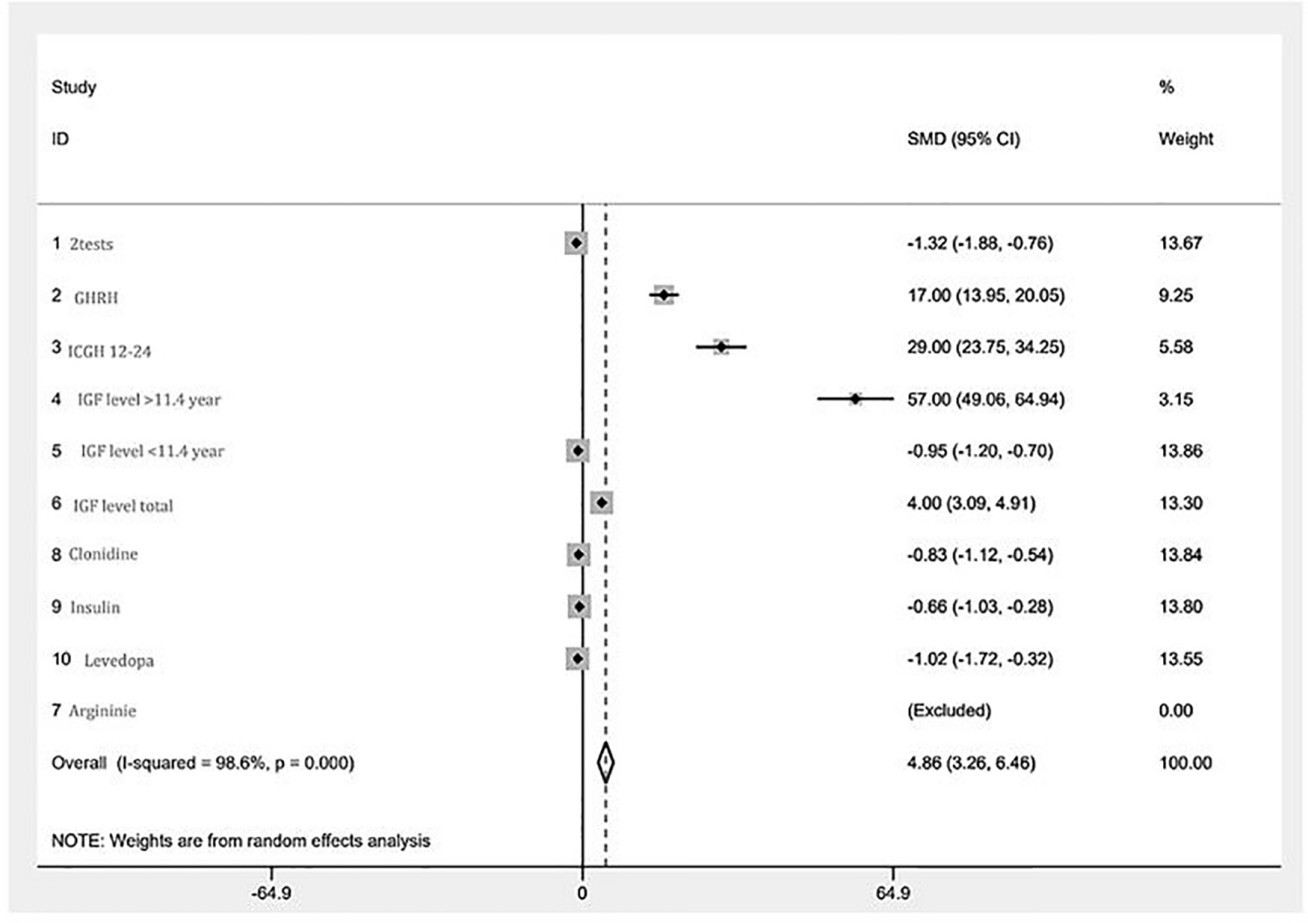
A forest plot of the GHRH-GH-IGF1 axis mini meta-analysis. Random effects meta-analysis of different tests. The right-hand column shows a plot of the measured weight of effect for each test type represented by a square, also incorporating confidence intervals represented by horizontal lines. The overall meta-analyzed measure of effect is represented on the plot as a dashed vertical line. The position of most points along the “0” line reflects the minimum degree of variance between the vast majority of tests.

A forest plot of standardized mean difference (SMD), and their 95% CI and weights for the different combined test results is presented in Figure 3. SMD expresses the size of the effect of the combined results for each test relative to the variability observed in the different studies in which that test was examined. Most of the results were found to be in close proximity to the overall meta-analyzed measure of effect, i.e., with a minimum degree of variance. One subgroup of IGF-1 levels is not in close proximity, but the overall cumulative IGF-1 level test does come within relative proximity. Two results are not in proximity: GHRH and ICGH 12–24.

Using random effect analysis, chi-squared heterogeneity was 567.47 with a *p*-value <0.001. I-squared was 98.6%. The estimate of between-study tau-squared variance was 4.862 with a *p*-value <0.001.

#### 3.2.1. Hypothalamic function

Hypothalamic function was assessed by 12-h (nocturnal) or 24-h integrated GH concentration and by GH stimulation tests that can be classified to “hypothalamus-mediated” and “pituitary-mediated”.

#### 3.2.2. Hypothalamus-mediated GH stimulation tests

**Figure 4:**
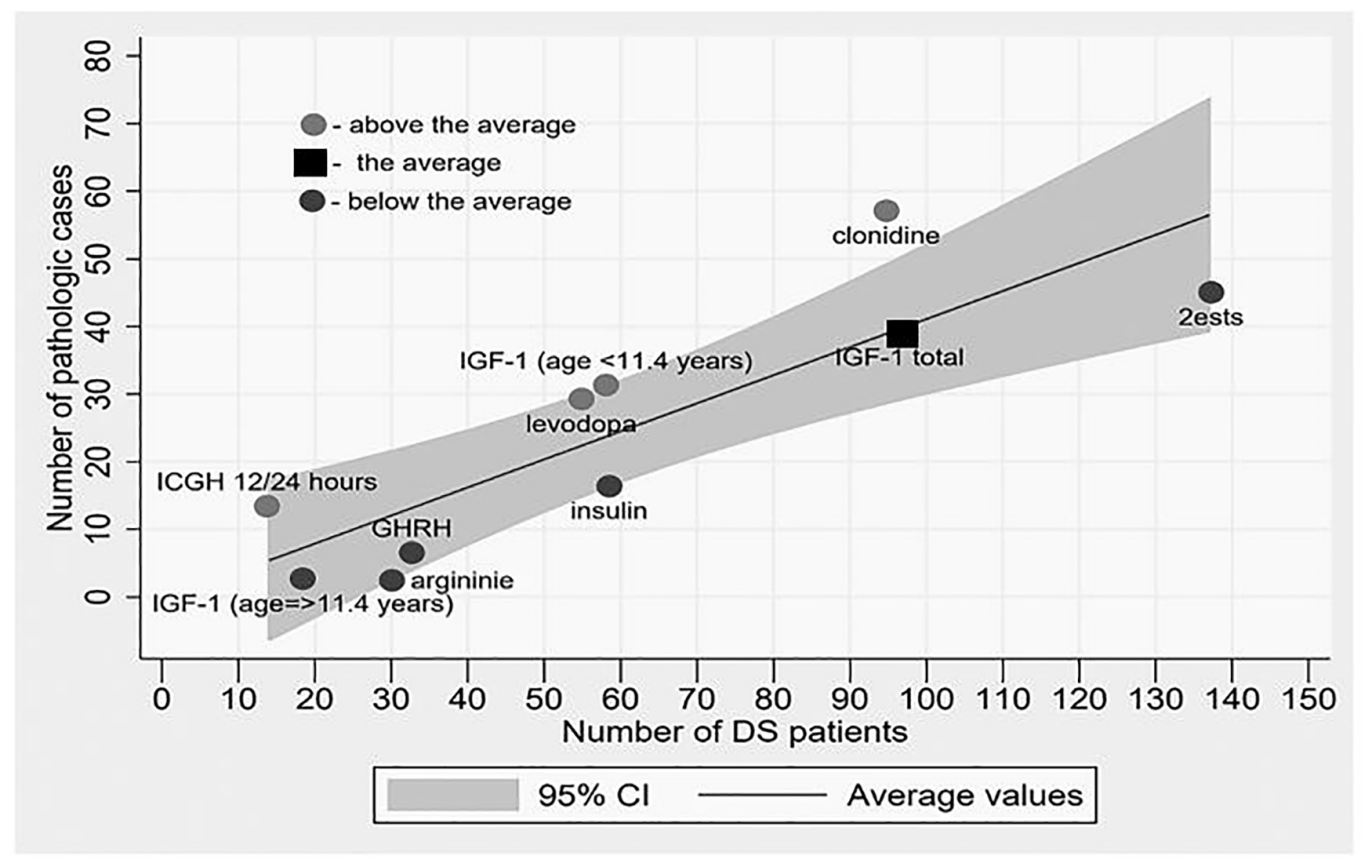
The degree of pathology results rate in each test relative to the weighted average of all tests. Dark dots represent “above the average” rate, light dots represent “beneath the average” rate and square represent “on average”. The potential for a pathological result in a particular test in a child with DS increases as one moves away from the mean line upwards.

Four such tests were conducted in DS patients. The mini meta-analysis of all the detailed and reported hypothalamus-mediated GH stimulation tests (8–10, 14–20, 22), reveals two main findings. Firstly, remarkable variability was observed between the results compared to the arginine test. The fraction of pathological results for the insulin tolerance test (17 of 59 children), levodopa test (29 of 55), and clonidine test (57 of 95) was found to be significantly higher than for the arginine test (2 of 30), with a *p-*value of <0.02. Figure 4. demonstrates the variance between different tests aimed at assessing the growth hormone axis in children with DS. Also, approximately one third of DS patients had two pathologic GH stimulation tests, and formally should be considered growth hormone deficient.

#### 3.2.3. GH neurosecretory dysfunction

The mini meta-analysis of all detailed reported 12-h (nocturnal) or 24-h integrated GH concentration reveals a very high (93%) pathological fraction (Figure 2, 4), indicating an almost universal defect among the DS population (15, 20, 23). From all included studies it was possible to extract data on just a single non-DS subject who underwent this test and in whose case the test came out normal, and therefore it is not possible to discuss the significance of the difference. Other nocturnal GH peak characteristics were also found to be significantly low compared to the control (23).

#### 3.2.4. Pituitary-mediated GH stimulation tests

GH releasing effect was studied in 12 DS patients showing that the response to hexarelin is normal and is similar to GHRH plus pyridostigmine, slightly higher than GHRH alone, and much higher than clonidine, levodopa, and insulin stimulation tests (22), with *p-*value of <0.0001, 0.000192, and 0.077, respectively.

### 3.3. GH receptor and bio-inactive GH

There was a difference between “endogenous” and “exogenous” IGF-1 generation tests. A normal reaction to the “exogenous” GH-induced IGF-1 generation test (12, 17), in the presence of an abnormal reaction to “endogenous” arginine induced IGF-1 generation test, indicates normal function of GH receptors and an “upward” or “proximal” problem. An indirect proof of the presence of GH receptor was obtained by the evaluation of the calculated bound GHBP (growth hormone binding protein)/ total GHBP ratio that was found to be normal and equal to controls and demonstrated their normal levels (17). On the other hand, the evaluation of the RRA/IRMA ratio revealed the presence of a reduced bioactivity of endogenous GH in some DS patients (17).

#### 3.3.1. IGF-1 synthesis and secretion -IGF-1 levels

The mini meta-analysis of the detailed and reported IGF-1 level in DS pediatric patients, according to an acceptable age-dependent normal range (13, 17, 19–21, 24–27), reveals that 41% are of low pathological level, 72% are under the 25th percentile, and 87% are under the 50th percentile. After splitting this group according to an age cut-off of 11.4 years, the results obtained are: 53% are of low pathological level, 81% are under the 25th percentile, and 98% are under the 50th percentile.

## 4. Discussion

The picture that emerges from this systematic review and mini meta-analysis is that the GHRH-GH-IGF1 axis is impaired in children with DS. There is a well-established impression of a hypothalamic impairment expressed in a combination of quantitative production and functional neurosecretion disorders. Apparently, a significant portion of the pathogenesis of short stature in children with DS is associated with impaired GHRH-GH-IGF1 axis function, as evident from the results of the various tests.

### 4.1. Hypothalamic dysfunction

A variety of findings supports the hypothesis that the hypothalamus plays a significant part in the short stature among children with DS (18), specifically the high rate of pathological results in the 12- or 24-hour integrated GH concentration test, the nocturnal GH peak characteristics and the differences between the various stimulation tests that act on different pathways.

#### 4.1.1. Hypothalamus-mediated GH stimulation tests

Insulin, levodopa, and clonidine tests revealed much more pathological results than the arginine test. This finding suggests involvement of the alpha-adrenergic neurotransmitter GHRH-mediating pathway and may point towards the original disturbance location.

#### 4.1.2. Pituitary-mediated GH stimulation tests

The significant difference between GHRH stimulating test results and ITT, Levodopa, Clonidine, and Arginine stimulation tests' results indicates normal or close to normal pituitary global function and supports hypothalamic dysfunction.

#### 4.1.3. GH neurosecretory dysfunction

The basis for understanding clinical disorders in the neuro-regulation of GH secretion is derived from the complexity of the CNS hypothalamic–pituitary axis. Studies in animals and humans demonstrate anatomic, physiological, and pharmacological evidence for neurosecretory control of GH secretion. The observation of a defect in the neuroregulatory control of GH secretion in CNS-irradiated humans and animals led to the hypothesis of a disorder in neurosecretion, GHND (growth hormone neurosecretion dysfunction), as a cause for short stature (28). The very high pathologic fraction result of the 12-h (nocturnal) or 24-h integrated GH concentration tests and nocturnal GH peak characteristics findings reflects a significant neurosecretory dysfunction.

### 4.2. IGF-1 synthesis and secretion -IGF-1 levels

DS subjects seem to lack the physiological switch from the production of fetal somatomedins and IGF-2 which are growth hormone independent, to IGF-1, which is growth hormone dependent (11). In light of the normal response of IGF-1 synthesis and secretion to “exogenous” GH, the abnormal findings might reflect a bio-inactive GH, an abnormality in the “endogenous” GH synthesis and secretion or action. A reduce RRA/IRMA ratio provides evidence of a discrepancy between GH binding to specific antibodies and to receptor. Such a finding has demonstrated GH molecular forms devoid of biological activity.

#### 4.2.1. IGF-1 receptor

Liver and brain IGF-1 receptors are normal in DS fetuses (29). The results in this review do not raise any suspicion of receptor interference.

### 4.3. Random effect analysis

A high heterogenicity index may be explained by the fact that, although the tested outcome- the rate of pathological tests- was uniform, this systematic review compares different tests that evaluate different “stations” along the hormonal axis. Nevertheless, it is possible to clearly identify the absolute majority of the results relatively close to the 0 line with the exception of 2 tests: GHRH and ICGH. In both cases, the total number of participants is relatively small compared to the other tests- a fact that increases the chance of sampling error. Although the total number of participants in those two tests is relatively small, this is the maximum number of participants reported in the literature to the best of our knowledge.

### 4.4. Conclusion

The findings of this review indicate a defect in three major components of the growth hormone axis in a significant proportion of pediatric DS patients. The first one is the quantitative capacity of the hypothalamus–pituitary axis, the second is the qualitative capacity of this axis, and the third is the reduced bioactivity of endogenous GH in some DS patients.

### 4.4.1. Implications for research and practice

The results of this review quite compellingly indicate that the GHRH-GH-IGF1 axis is impaired in children with DS and significantly strengthen the view that GHRH-GH-IGF1 axis assessment should be considered in short DS children.

According to our findings, assessing GHRH-GH-IGF1 axis function in DS children with the help of only classic stimulation tests may mislead the clinician. These tests mainly examine the quantitative capacity of growth hormone secretion. Although we have shown that about a third of children with DS suffer from an abnormal quantitative capacity and are formally diagnosed as having growth hormone deficiency based on two pathological stimulation tests, other disorders in the GHRH-GH-IGF1 axis such as GH neurosecretory dysfunction or bio-inactive GH are likely to be missed if we rely solely on growth hormone stimulation tests.

It is possible to consider performing a nocturnal spontaneous GH secretion test for evaluating the growth hormone axis in children with DS. This is a test that may sometimes be incorporated into clinical practice (30).

Exogenous growth hormone treatment is expected to provide a response to all three main issues identified in this study. In contrast to growth hormone receptor resistance and more distal disturbances such as IGF-1 receptor deficiency, the administration of proper recombinant growth hormone circumvents both the quantitative and qualitative disorders in the production and secretion of growth hormone, as well as defects in the endogenous protein structure. However, such therapy should undergo formal testing in prospective long term clinical studies.

## Data Availability

The original contributions presented in the study are included in the article/Supplementary Material, further inquiries can be directed to the corresponding author/s.

## References

[1] Presson AP Partyka G Jensen KM Devine OJ Rasmussen SA McCabe LL Current estimate of Down syndrome population prevalence in the United States. J Pediatr. (2013) 163:1163–8. 10.1016/j.jpeds.2013.06.013 23885965 PMC4445685

[2] Al-Biltagi MA. Epidemiology and prevalence of Down syndrome. In: Al-Biltagi MA, editor. Down syndrome children - an update. Bentham Science Publishers (2015). p. 3–44. 10.2174/97816810813421150101

[3] Cronk C Crocker AC Pueschel SM Shea AM Zackai E Pickens G Growth charts for children with Down syndrome: 1 month to 18 years of age. Pediatrics. (1988) 81:102–10. 10.1542/peds.81.1.102 2962062

[4] Cronk CE. Growth of children with Down’s syndrome: birth to age 3 years. Pediatrics. (1978) 61:564–8. 10.1542/peds.61.4.564 149290

[5] Moher D Liberati A Tetzlaff J Altman DG, PRISMA Group. Preferred reporting items for systematic reviews and meta-analyses: the PRISMA statement. PLoS Med. (2009) 6:e1000097. 10.1371/journal.pmed.1000097 19621072 PMC2707599

[6] Whiting P Savović J Higgins JPT Caldwell DM Reeves BC Shea B ROBIS: a new tool to assess risk of bias in systematic reviews was developed. J Clin Epidemiol. (2016) 69:225–34. 10.1016/j.jclinepi.2015.06.005 26092286 PMC4687950

[7] Higgins JPT Thompson SG Deeks JJ Altman DG. Measuring inconsistency in meta-analyses. Br Med J. (2003) 327:557–60. 10.1136/bmj.327.7414.557 12958120 PMC192859

[8] Milunsky A Lowy C Rubenstein AH Wright AD. Carbohydrate tolerance, growth hormone and insulin levels in mongolism. Dev Med Child Neurol. (1968) 10:25–31. 10.1111/j.1469-8749.1968.tb02833.x 4230643

[9] Pozsonyi J Friesen H. Growth hormone investigation in patients with mental dysfunction. Can Med Assoc J. (1971) 104:26–9. PMID: ; PMCID: 4250364 PMC1930822

[10] Ruvalcaba RH Thuline HC Kelley VC. Plasma growth hormone in patients with chromosomal anomalies. Arch Dis Child. (1972) 47:307–9. 10.1136/adc.47.252.307 5023484 PMC1648075

[11] Sara VR Gustavson KH Annerén G Hall K Wetterberg L. Somatomedins in Down’s syndrome. Biol Psychiatry. (1983) 18:803–11. PMID: 6225471

[12] Annerén G Sara VR Hall K Tuvemo T. Growth and somatomedin responses to growth hormone in Down’s syndrome. Arch Dis Child. (1986) 61:48–52. 10.1136/adc.61.1.48 2937371 PMC1777554

[13] Anneren G Gustavson K-H Sara VR Tuvemo T. Growth retardation in Down syndrome in relation to insulin-like growth factors and growth hormone. Am J Med Genet Suppl. (1990) 7:59–62. 10.1002/ajmg.1320370710 1963538

[14] Torrado C Bastian W Wisniewski KE Castells S. Treatment of children with Down syndrome and growth retardation with recombinant human growth hormone. J Pediatr. (1991) 119:478–83. 10.1016/s0022-3476(05)82068-2 1831841

[15] Castells S Torrado C Bastian W Wisniewski KE. Growth hormone deficiency in Down’s syndrome children. J Intellect Disabil Res. (1992) 36:29–43. 10.1111/j.1365-2788.1992.tb00469.x 1533556

[16] Pueschel SM. Growth hormone response after administration of L-dopa, clonidine, and growth hormone releasing hormone in children with Down syndrome. Res Dev Disabil. (1993) 14:291–8. 10.1016/0891-4222(93)90023-D 8210606

[17] Barreca A Rasore Quartino A Acutis MS Ponzani P Damonte G Miani E Assessment of growth hormone insulin like growth factor-I axis in Down’s syndrome. J Endocrinol Invest. (1994) 17:431–6. 10.1007/BF03347731 7930388

[18] Castells S Beaulieu I Torrado C Wisniewski KE Zarny S Gelato MC. Hypothalamic versus pituitary dysfunction in Down’s syndrome as cause of growth retardation. J Intellect Disabil Res. (1996) 40:509–17. 10.1111/j.1365-2788.1996.tb00661.x 9004111

[19] Castells S Abdel-Khalek IA Wisniewski KE. Long-term effects of recombinant human growth hormone on children with Down syndrome and growth retardation. Dev Brain Dysfunct. (1996) 9:144–57.

[20] Ragusa L Alberti A Proto C Romano C Colabucci F. Recombinant human growth hormone treatment in Down syndrome: the troina experience. Dev Brain Dysfunct. (1996) 9:158–64.

[21] Proto C Ragusa L Alberti A Romano C Colabucci F. Further data suggesting IGFBP-3 unreliability for the diagnosis of growth hormone deficiency in Down syndrome. Dev Brain Dysfunct. (1997) 10:15–9.

[22] Ragusa L Alberti A Romano C Proto C Bellone J Colabucci F Growth hormone releasing activity of hexarelin in Down syndrome. Dev Brain Dysfunct. (1996) 9:133–7.

[23] Ferri R Ragusa L Alberti A Elia M Musumeci SA Del Gracco S Growth hormone and sleep in Down syndrome. Dev Brain Dysfunct. (1996) 9:114–20.

[24] Arvat E Gianotti L Ragusa L Valetto MR Cappa M Aimaretti G The enhancing effect of pyridostigmine on the GH response to GHRH undergoes an accelerated age-related reduction in Down syndrome. Dementia. (1996) 7:288–92. 10.1159/000106894 8872421

[25] Ragusa L Valetto MR Proto C Alberti A Romano C Rossodivita A IGF-I levels in prepubertal and pubertal children with Down syndrome. Minerva Endocrinol. (1998) 23:31–6. PMID: 9844353

[26] Yasuhara A Yoshida Y. Beneficial effect of growth hormone on severe delay in motor development in a child with down syndrome. Clin Pediatr Endocrinol. (2001) 10:137–40. 10.1297/cpe.10.137

[27] El Gebali HH Zaky EA Agwa SH Mohamed EZ. Leptin, insulin like growth factor-1 and thyroid profile in a studied sample of Egyptian children with Down syndrome. Egypt J Med Hum Genet. (2014) 15:131–8. 10.1016/j.ejmhg.2014.01.007

[28] Bercu BB Diamond FB. Growth hormone neurosecretory dysfunction. Clin Endocrinol Metab. (1986) 15:537–90. 10.1016/s0300-595x(86)80010-x 2429794

[29] Sara VR Sjögren B Annerén G Gustavson KH Forsman A Hall K The presence of normal receptors for somatomedin and insulin in fetuses with Down’s syndrome. Biol Psychiatry. (1984) 19:591–8. PMID: 6329329

[30] Lennartsson O Nilsson O Lodefalk M. Discordance between stimulated and spontaneous growth hormone levels in short children is dependent on cut-off level and partly explained by refractoriness. Front Endocrinol. (2020) 11:584906. 10.3389/fendo.2020.584906 PMC7705110 33281744

